# *N*-(3-Chloro­benzo­yl)benzene­sulfonamide

**DOI:** 10.1107/S1600536809041051

**Published:** 2009-10-17

**Authors:** B. Thimme Gowda, Sabine Foro, P. A. Suchetan, Hartmut Fuess

**Affiliations:** a Department of Chemistry, Mangalore University, Mangalagangotri 574 199, Mangalore, India; b Institute of Materials Science, Darmstadt University of Technology, Petersenstrasse 23, D-64287 Darmstadt, Germany

## Abstract

In the crystal structure of the title compound, C_13_H_10_ClNO_3_S, the conformation of the N—H bond in the C—SO_2_—NH—C(O) segment is *anti* to the C=O bond. The dihedral angle between the two benzene rings is 87.5 (1)°. The crystal structure features inversion-related dimers linked by pairs of N—H⋯O(S) hydrogen bonds.

## Related literature

For background literature and similar structures, see: Gowda *et al.* (2008 ▶); Gowda, Foro, Nirmala *et al.* (2009 ▶); Gowda, Foro, Suchetan *et al.* (2009 ▶).
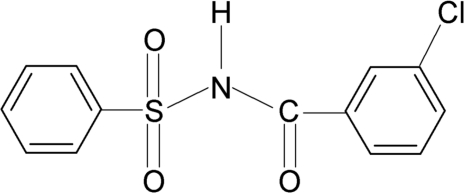


## Experimental

### 

#### Crystal data

C_13_H_10_ClNO_3_S
*M* *_r_* = 295.73
Monoclinic, 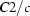

*a* = 21.309 (2) Å
*b* = 6.0953 (7) Å
*c* = 20.367 (2) Å
β = 92.48 (1)°
*V* = 2642.9 (5) Å^3^
*Z* = 8
Mo *K*α radiation
μ = 0.45 mm^−1^
*T* = 299 K
0.42 × 0.40 × 0.24 mm

#### Data collection

Oxford Diffraction Xcalibur diffractometer with a Sapphire CCD detector
Absorption correction: multi-scan (*CrysAlis RED*; Oxford Diffraction, 2009 ▶) *T*_min_ = 0.834, *T*_max_ = 0.900
5328 measured reflections
2714 independent reflections
1968 reflections with *I* > 2σ(*I*)
*R*_int_ = 0.015

#### Refinement

*R*[*F* ^2^ > 2σ(*F* ^2^)] = 0.039
*wR*(*F* ^2^) = 0.133
*S* = 0.92
2714 reflections
176 parameters
H atoms treated by a mixture of independent and constrained refinement
Δρ_max_ = 0.18 e Å^−3^
Δρ_min_ = −0.32 e Å^−3^

### 

Data collection: *CrysAlis CCD* (Oxford Diffraction, 2009 ▶); cell refinement: *CrysAlis RED* (Oxford Diffraction, 2009 ▶); data reduction: *CrysAlis RED*; program(s) used to solve structure: *SHELXS97* (Sheldrick, 2008 ▶); program(s) used to refine structure: *SHELXL97* (Sheldrick, 2008 ▶); molecular graphics: *PLATON* (Spek, 2009 ▶); software used to prepare material for publication: *SHELXL97*.

## Supplementary Material

Crystal structure: contains datablocks I, global. DOI: 10.1107/S1600536809041051/pk2198sup1.cif

Structure factors: contains datablocks I. DOI: 10.1107/S1600536809041051/pk2198Isup2.hkl

Additional supplementary materials:  crystallographic information; 3D view; checkCIF report

## Figures and Tables

**Table 1:** Hydrogen-bond geometry (Å, °)

*D*—H⋯*A*	*D*—H	H⋯*A*	*D*⋯*A*	*D*—H⋯*A*
N1—H1*N*⋯O1^i^	0.84 (2)	2.12 (2)	2.946 (2)	171 (2)

Symmetry code: (i) 
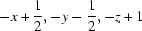
.

**Fig. 1.**
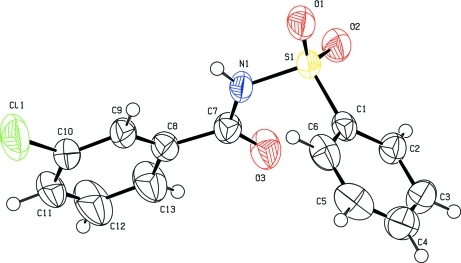
Molecular structure of (I), showing the atom labelling scheme and displacement ellipsoids (drawn at the 50% probability level).

**Fig. 2.**
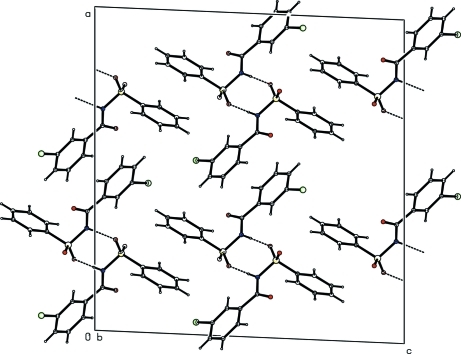
Molecular packing of (I) with hydrogen bonding shown as dashed lines.

## supplementary crystallographic information

### Comment

Diaryl acylsulfonamides are known as potent antitumor agents against a broad spectrum of human tumor xenografts (colon, lung, breast, ovary and prostate) in nude mice. As part of a study of the effect of ring and the side chain substituents on the crystal structures of *N*-aromatic sulfonamides (Gowda *et al.*, 2008; Gowda, Foro, Nirmala *et al.*, 2009; Gowda, Foro, Suchetan *et al.*, 2009), in the present work, the structure of *N*-(3-chlorobenzoyl)benzenesulfonamide (I) has been determined (Fig.1). The conformation of the of the N—H bond in the C—SO_2_—NH—C(O) segment of the structure is *anti* to the C=O bond, similar to that observed in *N*-(benzoyl)benzenesulfonamide (II) (Gowda, Foro, Suchetan *et al.*, 2009). Further, the C=O bond in the segment is *anti* to the *meta*-Cl in the benzoyl ring, while the conformation of the N—C bond in the C—SO_2_—NH—C(O) segment of the structure has "*gauche*" torsions with respect to the SO bonds. The molecule is twisted at the N atom with a dihedral angle of 89.9 (1)° between the sulfonyl benzene ring and the C—SO_2_—NH—C—O segment, compared to the value of 86.5 (1)° in (II). The dihedral angle between the two benzene rings is 87.5 (1)° in (I) and 80.3 (1)° in (II). The packing of molecules linked by pairs of N—H···O(S) hydrogen bonds (Table 1) is shown in Fig. 2.

### Experimental

The title compound was prepared by refluxing a mixture of 3-chlorobenzoic acid, benzene sulfonamide and phosphorous oxychloride for 5 h on a water bath. The resultant mixture was cooled and poured into ice cold water. The solid obtained was filtered, washed thoroughly with water and then dissolved in sodium bicarbonate solution.  The compound was later reprecipitated by acidifying the filtered solution with dilute HCl. The filtered and dried solid was recrystallized to the constant melting point.

Prism-like colourless single crystals of the title compound used in X-ray diffraction studies were obtained from a slow evaporation of its toluene solution at room temperature.

### Refinement

The H atom of the NH group was located in a difference map and and later restrained to N—H = 0.84 (2) Å. The other H atoms were positioned with idealized geometry using a riding model with C—H = 0.93 Å. All H atoms were refined with isotropic displacement parameters (set to 1.2 times of the *U*_eq_ of the parent atom).

### Crystal data

**Table:**

C_13_H_10_ClNO_3_S	*F*(000) = 1216
*M**_r_* = 295.73	*D*_x_ = 1.486 Mg m^−^^3^
Monoclinic, *C*2/*c*	Mo *K*α radiation, λ = 0.71073 Å
Hall symbol: -C 2yc	Cell parameters from 1936 reflections
*a* = 21.309 (2) Å	θ = 2.7–28.0°
*b* = 6.0953 (7) Å	µ = 0.45 mm^−^^1^
*c* = 20.367 (2) Å	*T* = 299 K
β = 92.48 (1)°	Prism, colourless
*V* = 2642.9 (5) Å^3^	0.42 × 0.40 × 0.24 mm
*Z* = 8	

### Data collection

**Table:**

Oxford Diffraction Xcalibur diffractometer with a Sapphire CCD detector	2714 independent reflections
Radiation source: fine-focus sealed tube	1968 reflections with *I* > 2σ(*I*)
graphite	*R*_int_ = 0.015
Rotation method data acquisition using ω and φ scans	θ_max_ = 26.4°, θ_min_ = 2.7°
Absorption correction: multi-scan (*CrysAlis RED*; Oxford Diffraction, 2009)	*h* = −26→16
*T*_min_ = 0.834, *T*_max_ = 0.900	*k* = −7→6
5328 measured reflections	*l* = −25→25

### Refinement

**Table:**

Refinement on *F*^2^	Secondary atom site location: difference Fourier map
Least-squares matrix: full	Hydrogen site location: inferred from neighbouring sites
*R*[*F*^2^ > 2σ(*F*^2^)] = 0.039	H atoms treated by a mixture of independent and constrained refinement
*wR*(*F*^2^) = 0.133	*w* = 1/[σ^2^(*F*_o_^2^) + (0.1*P*)^2^] where *P* = (*F*_o_^2^ + 2*F*_c_^2^)/3
*S* = 0.92	(Δ/σ)_max_ = 0.001
2714 reflections	Δρ_max_ = 0.18 e Å^−^^3^
176 parameters	Δρ_min_ = −0.32 e Å^−^^3^
0 restraints	Extinction correction: *SHELXL97* (Sheldrick, 2008), Fc^*^=kFc[1+0.001xFc^2^λ^3^/sin(2θ)]^-1/4^
Primary atom site location: structure-invariant direct methods	Extinction coefficient: 0.0038 (7)

### Special details

**Table:**

Experimental. CrysAlis RED (Oxford Diffraction, 2009) Empirical absorption correction using spherical harmonics, implemented in SCALE3 ABSPACK scaling algorithm.
Geometry. All e.s.d.'s (except the e.s.d. in the dihedral angle between two l.s. planes) are estimated using the full covariance matrix. The cell e.s.d.'s are taken into account individually in the estimation of e.s.d.'s in distances, angles and torsion angles; correlations between e.s.d.'s in cell parameters are only used when they are defined by crystal symmetry. An approximate (isotropic) treatment of cell e.s.d.'s is used for estimating e.s.d.'s involving l.s. planes.
Refinement. Refinement of *F*^2^ against ALL reflections. The weighted *R*-factor *wR* and goodness of fit *S* are based on *F*^2^, conventional *R*-factors *R* are based on *F*, with *F* set to zero for negative *F*^2^. The threshold expression of *F*^2^ > σ(*F*^2^) is used only for calculating *R*-factors(gt) *etc*. and is not relevant to the choice of reflections for refinement. *R*-factors based on *F*^2^ are statistically about twice as large as those based on *F*, and *R*- factors based on ALL data will be even larger.

### Fractional atomic coordinates and isotropic or equivalent isotropic displacement parameters (Å^2^)

**Table:**

	*x*	*y*	*z*	*U*_iso_*/*U*_eq_	
C1	0.20143 (9)	−0.0804 (3)	0.65509 (10)	0.0418 (5)	
C2	0.19836 (10)	0.0558 (4)	0.70922 (10)	0.0486 (5)	
H2	0.2161	0.1952	0.7087	0.058*	
C3	0.16863 (13)	−0.0179 (4)	0.76404 (12)	0.0648 (7)	
H3	0.1668	0.0720	0.8008	0.078*	
C4	0.14182 (12)	−0.2218 (5)	0.76472 (13)	0.0687 (7)	
H4	0.1218	−0.2697	0.8018	0.082*	
C5	0.14450 (12)	−0.3554 (4)	0.71097 (15)	0.0696 (7)	
H5	0.1257	−0.4929	0.7116	0.084*	
C6	0.17497 (11)	−0.2883 (3)	0.65551 (12)	0.0569 (6)	
H6	0.1776	−0.3808	0.6194	0.068*	
C7	0.13356 (10)	0.1419 (3)	0.52483 (10)	0.0483 (5)	
C8	0.08621 (9)	0.1089 (3)	0.46936 (10)	0.0486 (5)	
C9	0.08589 (10)	−0.0686 (4)	0.42751 (10)	0.0519 (5)	
H9	0.1175	−0.1737	0.4316	0.062*	
C10	0.03867 (11)	−0.0901 (4)	0.37955 (10)	0.0562 (6)	
C11	−0.00920 (12)	0.0610 (5)	0.37261 (13)	0.0705 (7)	
H11	−0.0411	0.0440	0.3404	0.085*	
C12	−0.00860 (15)	0.2364 (5)	0.41439 (18)	0.0949 (10)	
H12	−0.0404	0.3407	0.4102	0.114*	
C13	0.03849 (13)	0.2618 (4)	0.46282 (15)	0.0772 (8)	
H13	0.0380	0.3819	0.4910	0.093*	
N1	0.18539 (9)	0.0071 (3)	0.52611 (8)	0.0498 (5)	
H1N	0.1947 (11)	−0.082 (4)	0.4968 (12)	0.060*	
O1	0.28316 (7)	−0.1577 (3)	0.56683 (8)	0.0734 (6)	
O2	0.26351 (8)	0.2263 (3)	0.59692 (7)	0.0655 (5)	
O3	0.12636 (8)	0.2751 (3)	0.56792 (8)	0.0695 (5)	
Cl1	0.03840 (4)	−0.31286 (16)	0.32733 (4)	0.1016 (3)	
S1	0.24035 (2)	0.00980 (9)	0.58590 (2)	0.0490 (2)	

### Atomic displacement parameters (Å^2^)

**Table:**

	*U*^11^	*U*^22^	*U*^33^	*U*^12^	*U*^13^	*U*^23^
C1	0.0370 (10)	0.0454 (10)	0.0420 (10)	0.0088 (9)	−0.0084 (8)	−0.0040 (8)
C2	0.0523 (12)	0.0503 (12)	0.0429 (11)	−0.0007 (9)	−0.0009 (9)	−0.0076 (9)
C3	0.0667 (16)	0.0830 (18)	0.0449 (12)	0.0055 (13)	0.0039 (11)	−0.0048 (12)
C4	0.0602 (16)	0.0855 (18)	0.0602 (15)	−0.0003 (13)	0.0010 (12)	0.0214 (14)
C5	0.0590 (15)	0.0527 (14)	0.096 (2)	−0.0034 (11)	−0.0082 (15)	0.0199 (14)
C6	0.0538 (13)	0.0464 (12)	0.0690 (15)	0.0067 (10)	−0.0144 (11)	−0.0094 (11)
C7	0.0480 (12)	0.0527 (12)	0.0437 (11)	0.0083 (10)	−0.0030 (9)	−0.0027 (9)
C8	0.0418 (11)	0.0587 (13)	0.0447 (11)	0.0093 (10)	−0.0036 (9)	−0.0001 (10)
C9	0.0435 (12)	0.0708 (14)	0.0408 (11)	0.0110 (10)	−0.0050 (9)	−0.0031 (10)
C10	0.0480 (13)	0.0786 (15)	0.0416 (11)	−0.0027 (12)	−0.0042 (9)	0.0001 (11)
C11	0.0496 (14)	0.098 (2)	0.0621 (15)	0.0053 (14)	−0.0201 (12)	0.0098 (14)
C12	0.0680 (18)	0.097 (2)	0.117 (3)	0.0362 (16)	−0.0345 (18)	−0.009 (2)
C13	0.0631 (16)	0.0783 (17)	0.0880 (18)	0.0275 (14)	−0.0211 (14)	−0.0190 (15)
N1	0.0451 (10)	0.0688 (12)	0.0348 (9)	0.0139 (8)	−0.0067 (7)	−0.0155 (8)
O1	0.0482 (9)	0.1156 (15)	0.0554 (10)	0.0295 (9)	−0.0097 (8)	−0.0298 (10)
O2	0.0635 (10)	0.0830 (11)	0.0497 (9)	−0.0240 (9)	−0.0011 (8)	−0.0036 (8)
O3	0.0721 (11)	0.0670 (10)	0.0680 (11)	0.0201 (9)	−0.0139 (9)	−0.0271 (9)
Cl1	0.0912 (6)	0.1320 (7)	0.0789 (5)	0.0054 (5)	−0.0282 (4)	−0.0466 (5)
S1	0.0378 (3)	0.0713 (4)	0.0374 (3)	0.0049 (2)	−0.0044 (2)	−0.0125 (2)

### Geometric parameters (Å, °)

**Table:**

C1—C2	1.384 (3)	C8—C9	1.377 (3)
C1—C6	1.387 (3)	C8—C13	1.382 (3)
C1—S1	1.754 (2)	C9—C10	1.378 (3)
C2—C3	1.382 (3)	C9—H9	0.9300
C2—H2	0.9300	C10—C11	1.377 (4)
C3—C4	1.369 (3)	C10—Cl1	1.725 (2)
C3—H3	0.9300	C11—C12	1.366 (4)
C4—C5	1.367 (4)	C11—H11	0.9300
C4—H4	0.9300	C12—C13	1.385 (4)
C5—C6	1.389 (4)	C12—H12	0.9300
C5—H5	0.9300	C13—H13	0.9300
C6—H6	0.9300	N1—S1	1.6527 (18)
C7—O3	1.210 (2)	N1—H1N	0.84 (2)
C7—N1	1.376 (3)	O1—S1	1.4340 (16)
C7—C8	1.495 (3)	O2—S1	1.4232 (16)
			
C2—C1—C6	120.7 (2)	C8—C9—C10	119.8 (2)
C2—C1—S1	119.47 (17)	C8—C9—H9	120.1
C6—C1—S1	119.85 (16)	C10—C9—H9	120.1
C3—C2—C1	119.2 (2)	C11—C10—C9	121.6 (2)
C3—C2—H2	120.4	C11—C10—Cl1	118.72 (19)
C1—C2—H2	120.4	C9—C10—Cl1	119.64 (18)
C4—C3—C2	120.6 (2)	C12—C11—C10	118.2 (2)
C4—C3—H3	119.7	C12—C11—H11	120.9
C2—C3—H3	119.7	C10—C11—H11	120.9
C5—C4—C3	120.1 (2)	C11—C12—C13	121.2 (3)
C5—C4—H4	120.0	C11—C12—H12	119.4
C3—C4—H4	120.0	C13—C12—H12	119.4
C4—C5—C6	120.9 (2)	C8—C13—C12	120.0 (3)
C4—C5—H5	119.6	C8—C13—H13	120.0
C6—C5—H5	119.6	C12—C13—H13	120.0
C1—C6—C5	118.6 (2)	C7—N1—S1	123.41 (15)
C1—C6—H6	120.7	C7—N1—H1N	126.0 (17)
C5—C6—H6	120.7	S1—N1—H1N	110.5 (17)
O3—C7—N1	120.9 (2)	O2—S1—O1	118.86 (11)
O3—C7—C8	122.38 (19)	O2—S1—N1	110.81 (10)
N1—C7—C8	116.66 (17)	O1—S1—N1	103.42 (9)
C9—C8—C13	119.1 (2)	O2—S1—C1	109.68 (9)
C9—C8—C7	123.94 (18)	O1—S1—C1	108.83 (11)
C13—C8—C7	116.8 (2)	N1—S1—C1	104.13 (10)
			
C6—C1—C2—C3	0.1 (3)	Cl1—C10—C11—C12	179.8 (2)
S1—C1—C2—C3	−178.81 (18)	C10—C11—C12—C13	−0.5 (5)
C1—C2—C3—C4	−0.8 (4)	C9—C8—C13—C12	−0.5 (5)
C2—C3—C4—C5	0.3 (4)	C7—C8—C13—C12	−177.1 (3)
C3—C4—C5—C6	0.9 (4)	C11—C12—C13—C8	0.4 (5)
C2—C1—C6—C5	1.0 (3)	O3—C7—N1—S1	1.5 (3)
S1—C1—C6—C5	179.95 (17)	C8—C7—N1—S1	−176.23 (15)
C4—C5—C6—C1	−1.5 (3)	C7—N1—S1—O2	−52.6 (2)
O3—C7—C8—C9	−165.9 (2)	C7—N1—S1—O1	178.97 (19)
N1—C7—C8—C9	11.8 (3)	C7—N1—S1—C1	65.3 (2)
O3—C7—C8—C13	10.6 (4)	C2—C1—S1—O2	−0.9 (2)
N1—C7—C8—C13	−171.7 (2)	C6—C1—S1—O2	−179.87 (16)
C13—C8—C9—C10	0.7 (4)	C2—C1—S1—O1	130.64 (17)
C7—C8—C9—C10	177.1 (2)	C6—C1—S1—O1	−48.30 (19)
C8—C9—C10—C11	−0.8 (4)	C2—C1—S1—N1	−119.56 (17)
C8—C9—C10—Cl1	−179.90 (18)	C6—C1—S1—N1	61.49 (18)
C9—C10—C11—C12	0.7 (4)		

### Hydrogen-bond geometry (Å, °)

**Table:**

*D*—H···*A*	*D*—H	H···*A*	*D*···*A*	*D*—H···*A*
N1—H1N···O1^i^	0.84 (2)	2.12 (2)	2.946 (2)	171 (2)

Symmetry codes: (i) −*x*+1/2, −*y*−1/2, −*z*+1.
